# Comparison of Older Adult and Healthcare Provider Beliefs About Fall Prevention Strategies

**DOI:** 10.1093/geroni/igab046.3372

**Published:** 2021-12-17

**Authors:** Gwen Bergen, Ankita Henry, Yara Haddad

**Affiliations:** Centers for Disease Control and Prevention (CDC), Atlanta, Georgia, United States

## Abstract

Older adults reported about 36 million falls in 2018. Although effective strategies are available to minimize fall risk, little is known about older adults’ and healthcare providers’ awareness of these strategies. This study describes and compares older adults’ and healthcare providers’ beliefs about fall prevention strategies. Demographic and fall-related data for older adults were obtained from the 2019 fall cohort of Porter Novelli ConsumerStyles. Similar data from primary care practitioners, nurse practitioners, and physician assistants, were gathered from the 2019 cohort of DocStyles. Percentages and 95% confidence intervals were calculated to compare older adults and providers. Most providers (91.3%) and older adults (85.1%) believed falls can be prevented. High percentages of providers and older adults identified strength and balance exercises (90.7% and 82.8% respectively) and making homes safer (90.5% and 79.9% respectively) as strategies that help prevent falls. More providers reported that Tai Chi (45.7%) and managing medications (84.2%) can prevent falls compared to older adults (21.7% and 24.0% respectively; p<0.0001). Sizable percentages of providers and older adults endorsed less evidence-based strategies including aerobic exercise (70.7% and 58.4% respectively) and being more careful (69.3% and 81.6% respectively). Among older adults, lower endorsement of evidence-based strategies (e.g., Tai Chi, medication management) coupled with higher endorsement of limited evidence-based strategies (e.g., being careful, aerobic exercise) suggest some older adults lack awareness of effective fall prevention interventions. Increased patient and provider communication can increase awareness about the benefits of evidence-based strategies for fall prevention.

